# Supplementary figures and images for: An automated homecage system for multiwhisker detection and discrimination learning in mice
Source: PLoS One. 2020 Dec 2;15(12):e0232916. doi: 10.1371/journal.pone.0232916 (PMC7710058; doi:10.1371/journal.pone.0232916)

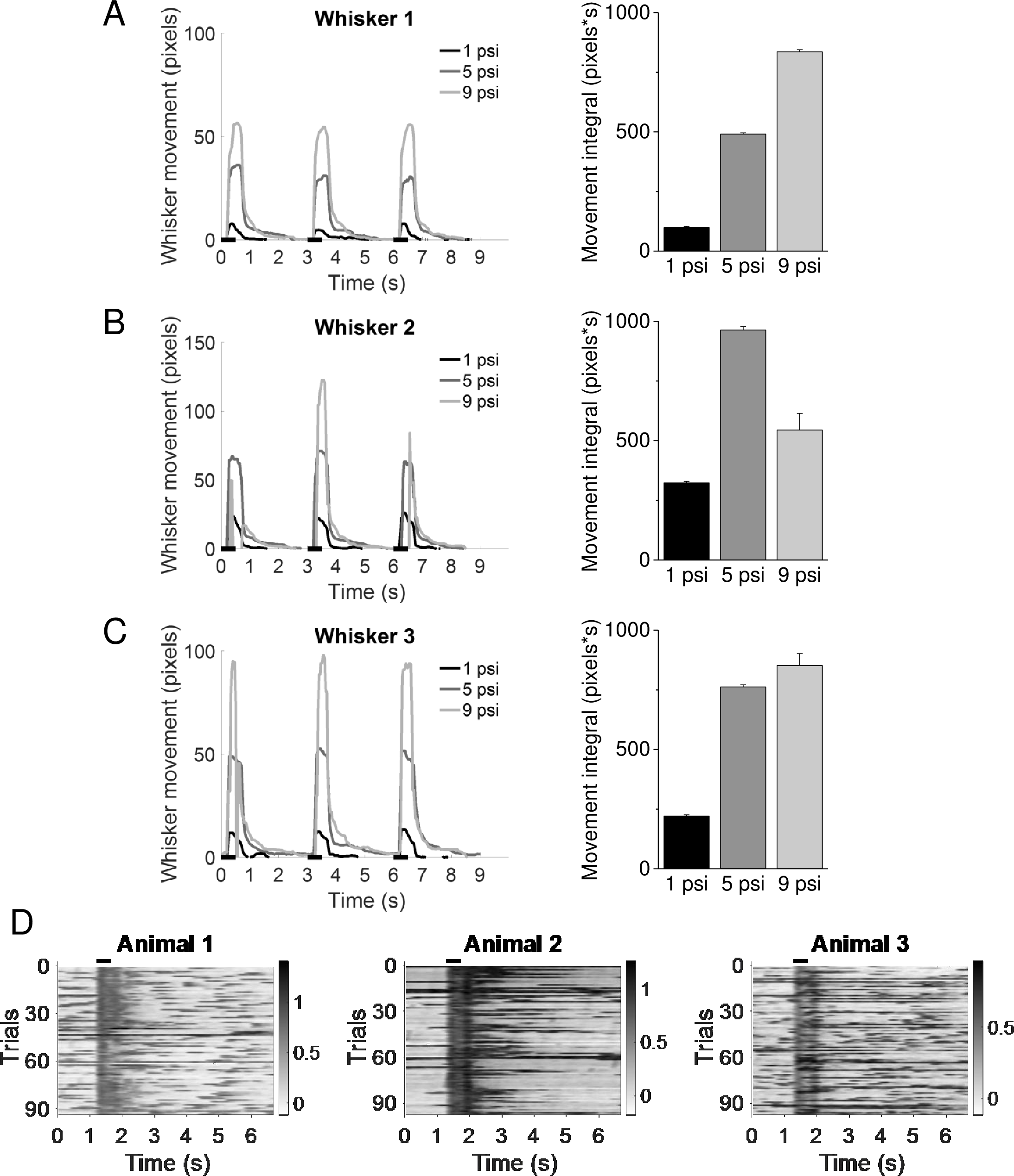

Supplement: S1 Fig — A-C) Left, example movement traces of the three whiskers indicated in Fig 1A at 3 different air puff strengths. Black bars indicate air puff (500 ms). Right, average movement across 50 trials at 3 different air puff strengths. D) Normalized whisker motion energy (arbitrary unit, see methods) before and after air puff stimulus (black bar at the top) in three animals. ~100 trials per animal. (TIF) [file pone.0232916.s005.tif]

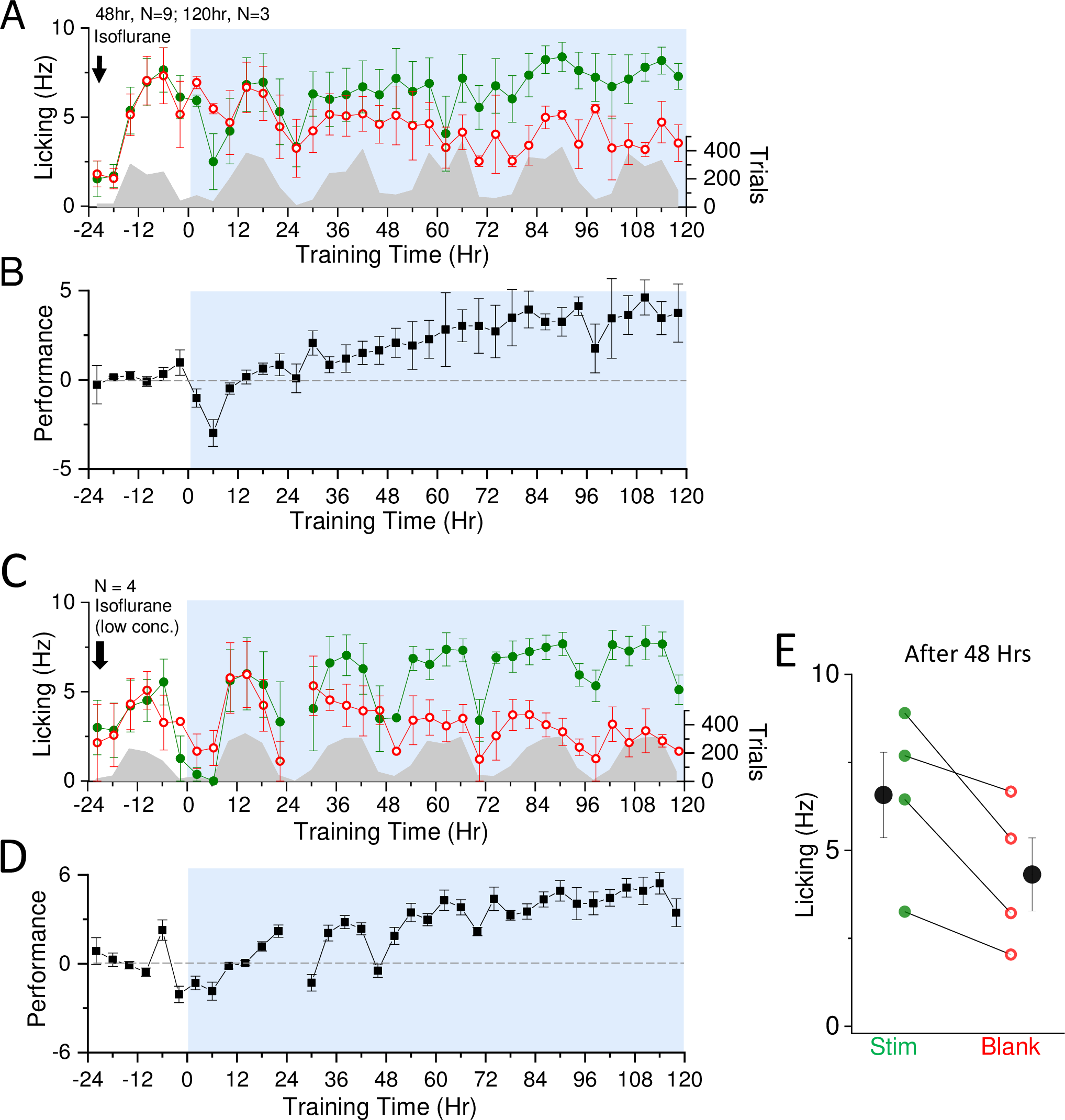

Supplement: S2 Fig — A) Isoflurane-exposed mice were placed in training chamber for acclimation period followed by training. Air puff set at 6 psi and association training is indicated at t = 0 (12 noon/daylight period). Mean lick frequency for water delivery (green) or blank (red) trials, binned at 4 hr intervals, is overlaid upon mean number of initiated trials (grey) across training days. B) Mean performance (lick frequency for water trials—lick frequency for blank trials) for each 4-hour bin during the acclimation period (-24 to 0 hr) and training phases (0 to 120 hrs). C) Same as in (A) but with low-concentration isoflurane (3%) exposure. D) Same as in (B) but with low-concentration isoflurane (3%) exposure. E) Mean lick frequency for the last 20% of total trials for each low-isoflurane induced animal exposed to 6 psi intensity air puff. N = 4 animals. (TIF) [file pone.0232916.s006.tif]

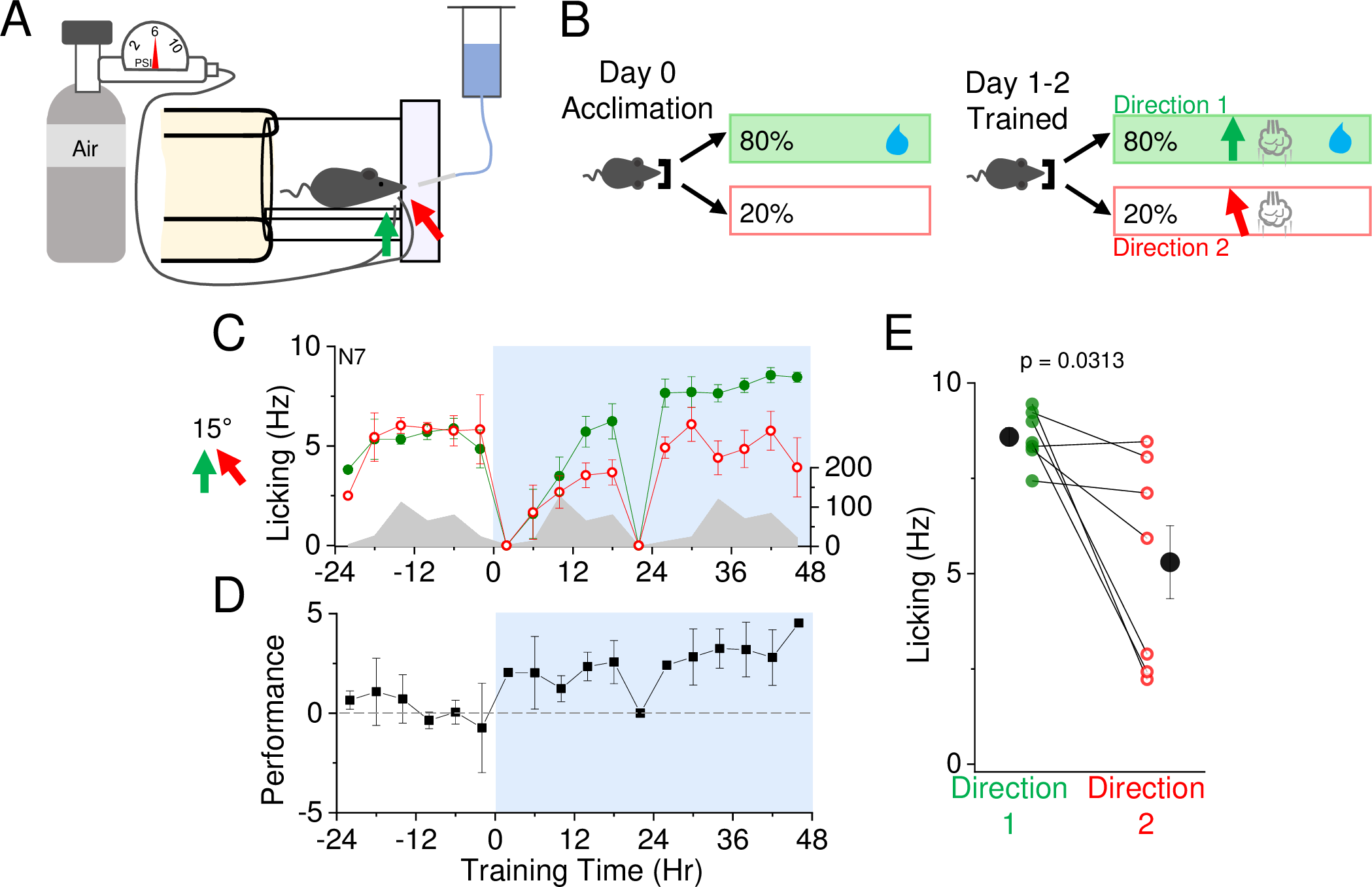

Supplement: S3 Fig — A) Profile view of bidirectional air puff training apparatus. B) Reward contingencies during training. Left, during the initial 24 hour acclimation period, animals receive water on 80% of initiated trials with no air puff. Right, during the training period, animals receive water and an air puff from one direction on 80% of initiated trials, and an air puff from a different direction without water on the remaining 20% of trials. C) Mean lick frequency of animals exposed to air puffs 15 degrees apart for water and blank trials, binned at 4 hr intervals. Air puff association training is indicated at t = 0 (12 noon/daylight period). Mean lick frequency for water delivery (green) or blank (red) trials is overlaid upon mean number of initiated trials (grey) across training days. D) Mean performance (lick frequency for water trials—lick frequency for blank trials) for each 4 hour bin during the acclimation period (-24 to 0 hr) and training phases with air puffs 15 degrees apart (0 to 48 hrs). E) Mean lick frequency of each animal exposed to air puffs 15 degrees apart for the last 20% of total trials. N = 7 animals. (TIF) [file pone.0232916.s007.tif]
