# Supplementary material for: An automated homecage system for multiwhisker detection and discrimination learning in mice
Source: PLoS One. 2020 Dec 2;15(12):e0232916. doi: 10.1371/journal.pone.0232916 (PMC7710058; doi:10.1371/journal.pone.0232916)
Supplement: S1 Table — Performance values of the last 20% of total trials at 48 hours for each animal. Performance values across conditions were compared using the Kruskal-Wallis test for non-normally distributed data, followed by a Dunn-Sidak post-hoc test to compare performance values for selected groups of animals. (DOCX) [file pone.0232916.s008.docx]

S1 Table. Performance values for individual animals arranged by experimental group with associated statistical tests

| **CONDITION** |  |  |  |  |  |  |  |  |  |  |  |  |  |  |  |  |  |
| --- | --- | --- | --- | --- | --- | --- | --- | --- | --- | --- | --- | --- | --- | --- | --- | --- | --- |
| **Intensity** |  |  |  | **Pretreat** |  |  |  |  | **Reward** |  |  |  | **Direction** |  |  |  |  |
| *6 psi* | *1-2 psi* | *9 psi* |  | *6 psi* | *Low Iso* | *High Iso* | *Barbered* |  | *6 psi* | *Aspartame* | *50/50* |  | *6 psi* | *5 deg* | *15 deg* | *180 deg* |  |
|  |  |  |  |  |  |  |  |  |  |  |  |  |  |  |  |  |  |
| -0.62319 | 3.77976 | 2.24444 |  | -0.62319 | 1.22054 | -0.38889 | 1.12836 |  | -0.62319 | 1.23398 | 7.03431 |  | -0.62319 | 3.98955 | 1.34085 | 1.27252 |  |
| 3.97996 | 1.21212 | 2.12121 |  | 3.97996 | 1.02273 | -0.37577 | -3.50224 |  | 3.97996 | 3.22431 | 7.05546 |  | 3.97996 | 2.44444 | 0.81512 | 5.74786 |  |
| 0.88949 | -0.93145 | 0.68056 |  | 0.88949 | 3.56452 | -0.42484 | -3.34167 |  | 0.88949 | 2.08333 | 1.91558 |  | 0.88949 | 2.63889 | 5.15152 | 0 |  |
| 0.45718 | 2.4263 | 1.27778 |  | 0.45718 | 3.23168 | -0.0614 | -2.56206 |  | 0.45718 | -0.67155 | -0.24155 |  | 0.45718 | 2.88462 | 4.8062 | -1.50794 |  |
| -1.66667 | 0.15278 | 0.30303 |  | -1.66667 |  | -0.83857 | -3.19396 |  | -1.66667 | 2.14434 | -0.05423 |  | -1.66667 | 2.79609 | 6.98925 | 0.37241 |  |
| 3.6828 | -0.35165 | -0.07937 |  | 3.6828 |  | 2.01832 | -0.89912 |  | 3.6828 | 0.68273 | -0.08761 |  | 3.6828 | 2.94241 | -1.16422 | 1.16239 |  |
| 0.68424 | 5.07937 | 4.93789 |  | 0.68424 |  | -2.02822 | 1.15344 |  | 0.68424 | 5.33334 | 1.80776 |  | 0.68424 |  | 0.53257 | -0.94931 |  |
| 1.53086 | 0.42424 |  |  | 1.53086 |  | -3.0681 | 0.7672 |  | 1.53086 | 4.35574 | 3.85831 |  | 1.53086 |  | -0.20833 | 1.92453 |  |
| 3.6828 | 2.29167 |  |  | 3.6828 |  | -0.84665 | 2.16524 |  | 3.6828 | 0.95076 | 0.45948 |  | 3.6828 |  | 7.50863 | 1.53419 |  |
| 1.38651 | -0.1473 |  |  | 1.38651 |  |  |  |  | 1.38651 |  | 5.0471 |  | 1.38651 |  |  |  |  |
| 3.82716 | -0.67766 |  |  | 3.82716 |  |  |  |  | 3.82716 |  | 2.06527 |  | 3.82716 |  |  |  |  |
| -0.22876 | 0.89027 |  |  | -0.22876 |  |  |  |  | -0.22876 |  |  |  | -0.22876 |  |  |  |  |
| -0.95176 | 5.88095 |  |  | -0.95176 |  |  |  |  | -0.95176 |  |  |  | -0.95176 |  |  |  |  |
| 2.58065 | 3.73756 |  |  | 2.58065 |  |  |  |  | 2.58065 |  |  |  | 2.58065 |  |  |  |  |
| 3.90783 | -0.88803 |  |  | 3.90783 |  |  |  |  | 3.90783 |  |  |  | 3.90783 |  |  |  |  |
|  | -1.66667 |  |  |  |  |  |  |  |  |  |  |  |  |  |  |  |  |
|  | -0.21663 |  |  |  |  |  |  |  |  |  |  |  |  |  |  |  |  |
|  | 1.26126 |  |  |  |  |  |  |  |  |  |  |  |  |  |  |  |  |
|  | 0.60317 |  |  |  |  |  |  |  |  |  |  |  |  |  |  |  |  |
|  | -0.46429 |  |  |  |  |  |  |  |  |  |  |  |  |  |  |  |  |
|  | -0.23658 |  |  |  |  |  |  |  |  |  |  |  |  |  |  |  |  |
|  | |  |  |  | |  |  |  |  | |  |  |  | |  |  |  |
| **STATISTICAL TESTS** | |  |  |  | |  |  |  |  | |  |  |  | |  |  |  |
| **Intensity** | |  |  | **Pretreat** | |  |  |  | **Reward** | |  |  | **Direction** | |  |  |  |
| ***Kruskal-Wallis*** | |  |  | ***Kruskal-Wallis*** | |  |  |  | ***Kruskal-Wallis*** | |  |  | ***Kruskal-Wallis*** | |  |  |  |
| P=0.47 |  |  |  | P=0.01 |  |  |  |  | P=0.61 |  |  |  | P=0.21 |  |  |  |  |
|  |  |  |  | ***Dunn-Sidak post-hoc*** | | ***P*** |  |  |  |  |  |  |  |  |  |  |  |
|  |  |  |  | 6 psi | Low Iso | 0.99 |  |  |  |  |  |  |  |  |  |  |  |
|  |  |  |  | 6 psi | High Iso | 0.10 |  |  |  |  |  |  |  |  |  |  |  |
|  |  |  |  | 6 psi | Barbered | 0.09 |  |  |  |  |  |  |  |  |  |  |  |
|  |  |  |  | Low Iso | High Iso | 0.15 |  |  |  |  |  |  |  |  |  |  |  |
|  |  |  |  | Low Iso | Barbered | 0.14 |  |  |  |  |  |  |  |  |  |  |  |
|  |  |  |  | High Iso | Barbered | 1.00 |  |  |  |  |  |  |  |  |  |  |  |
